# Development and validation of fear of hypoglycemia screener: results from the T1D exchange registry

**DOI:** 10.1186/s41687-023-00585-9

**Published:** 2023-05-09

**Authors:** Jingwen Liu, Jiat-Ling Poon, Jeoffrey Bispham, Magaly Perez-Nieves, Allyson Hughes, Katherine Chapman, Beth Mitchell, Korey Hood, Frank Snoek, Lawrence Fisher

**Affiliations:** 1 T1D Exchange, Boston, MA USA; 2 Eli Lilly and Company, Lilly Corporate Center, Indianapolis, IN 46285 USA; 3 Stanford University School of Medicine, Stanford, CA USA; 4 Department of Medical Psychology Amsterdam University Medical Centres, Vrije Universiteit Amsterdam, Amsterdam, The Netherlands; 5 Department of Family and Community Medicine, University of California San Francisco, San Francisco, CA USA

**Keywords:** Behavior scale, Fear of hypoglycemia, T1D, Worry scale

## Abstract

**Background:**

Fear of Hypoglycemia (FoH) in people with diabetes has a significant impact on their quality of life, psychological well-being, and self-management of disease. There are a few questionnaires assessing FoH in people living with diabetes, but they are more often used in research than clinical practice. This study aimed to develop and validate a short and actionable FoH screener for adults living with type 1 diabetes (T1D) for use in routine clinical practice.

**Methods:**

We developed an initial screener based on literature review and, interviews with healthcare providers (HCPs) and people with T1D. We developed a cross-sectional web-based survey, which was then conducted to examine the reliability and validity of the screener. Adults (aged ≥ 18 years) with diagnosis of T1D for ≥ 1 year were recruited from the T1D Exchange Registry (August–September 2020). The validation analyses were conducted using exploratory factor analyses, correlation, and multivariable regression models for predicting cut-off scores for the final screener.

**Results:**

The final FoH screener comprised nine items assessing two domains, “worry” (6-items) and “avoidance behavior” (three items), in 592 participants. The FoH screener showed good internal consistency (Cronbach’s α = 0.88). The screener also demonstrated high correlations (r = 0.71–0.75) with the Hypoglycemia Fear Survey and moderate correlations with depression, anxiety, and diabetes distress scales (r = 0.44–0.66). Multivariable regression analysis showed that higher FoH screener scores were significantly associated with higher glycated hemoglobin (HbA1c) (b = 0.04) and number of comorbidities (b = 0.03).

**Conclusions:**

This short FoH screener demonstrated good reliability and validity. Further research is planned to assess clinical usability to identify patients with FoH and assist effective HCP-patient conversations.

**Supplementary Information:**

The online version contains supplementary material available at 10.1186/s41687-023-00585-9.

## Background

People with diabetes frequently experience anxiety and stress-related disorders (e.g., generalized anxiety disorder [GAD], specific phobias, and posttraumatic stress disorder [PTSD]) [1, 2]. Among diabetes-related anxieties, fear of hypoglycemia (FoH) is defined as an ‘extreme worry or anxiety about low blood glucose and its consequences’ is prevalent in people with diabetes [3]. It has a significant impact on individuals’ quality of life, psychological state, and management of disease [4–7]. The American Diabetes Association’s (ADA) position statement on psychosocial care emphasizes the need for screening for FoH using standardized and validated tools [1]. ADA recommends referring people with diabetes who have a positive screen for elevated FoH to behavioral or mental health providers for evaluation and treatment [3].

In the past decades, a number of questionnaires have been developed to capture FoH in people living with diabetes: the Hypoglycemia Fear Survey-II (HFS-II); the Hypoglycemic Confidence Scale (HCS); the Hypoglycemic Attitudes and Behavior Scale (HABS); the Fear of Hypoglycemia Scale (FH-15); and the Quick Screening for Fear of Hypoglycemia Instrument (QSFH) [8–13]. These scales tap into fear/worry around (severe) hypoglycemia, confidence in managing hypoglycemia, and behaviors aimed to avoid hypoglycemia. Though some of these instruments are used as outcome measures in research settings, they are not commonly used as a screening tool in clinical practice [3]. The existing FoH tools are used primarily for research purposes. Often these instruments lack in providing clear cut-off scores, which limits their usefulness in clinical settings [14, 15].

There is still a need for practical screening tools that can flag problematic anxiety around hypoglycemia and quickly guide healthcare providers (HCPs) to areas requiring attention for additional management and/or diabetes education in clinical practice [13, 16].

This study aimed to develop and validate a short and actionable screening tool for HCPs to use in routine practice and to identify adults with type 1 diabetes (T1D) who need treatment and/or additional diabetes education around hypoglycemia management, in accordance with the ADA’s position statement [1, 3].

## Materials and methods

### Development phase

**Fig. 1:**
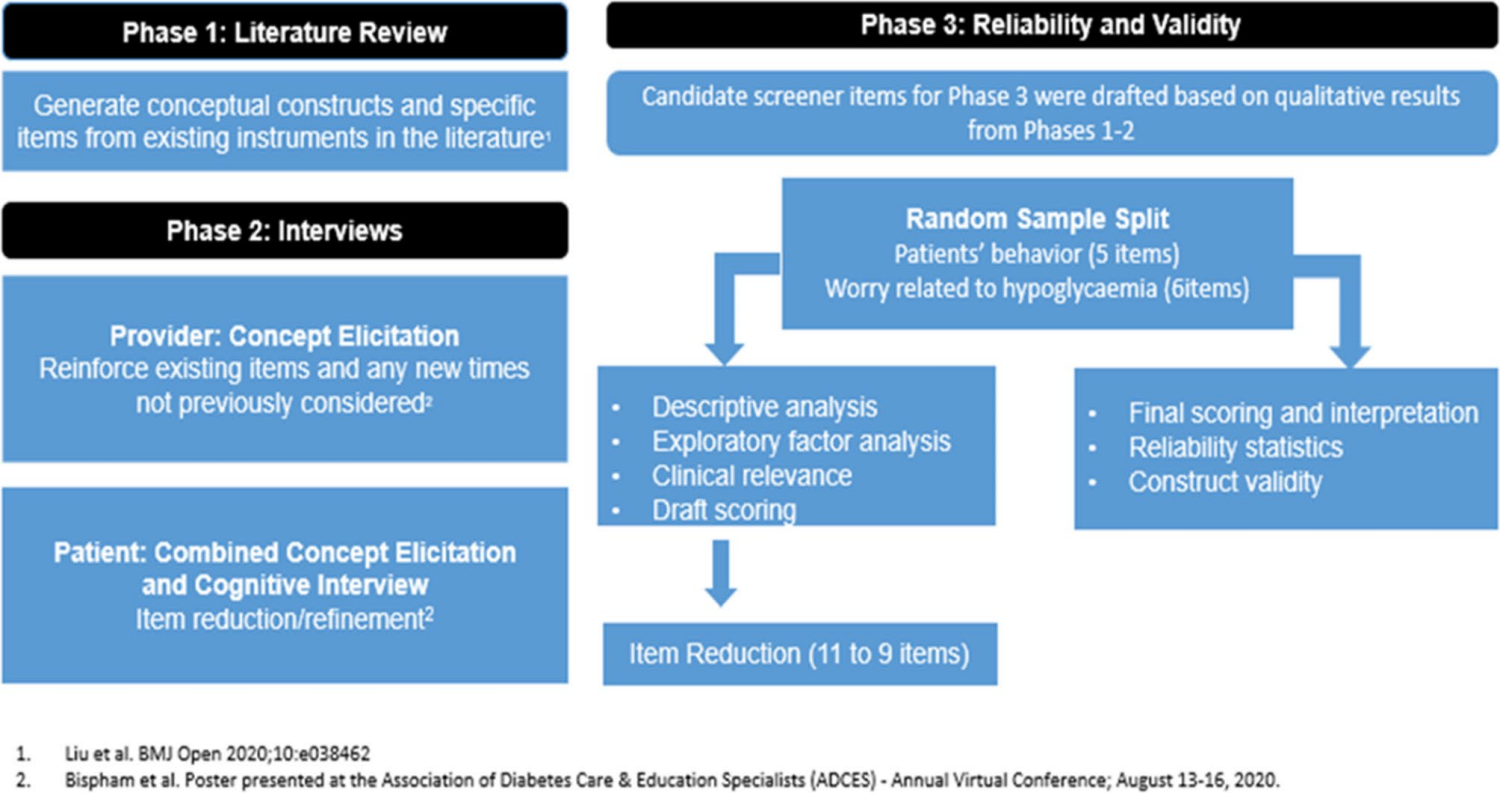
Study Design

In the initial phase of the study, we conducted a literature review to summarize and identify key constructs associated with FoH and existing measures assessing FoH (Fig. 1). Semi-structured interviews were then conducted with ten HCPs (endocrinologists N = 6, certified diabetes educators N = 4) [17] to confirm the constructs identified in the review, identify potential new constructs, and understand current clinical practice assessing and treating FoH [17, 18]. Based on these steps, an initial pool of 23 items (Supplementary Table 1) was drafted for the FoH screener and debriefed in cognitive interviews with 22 adults living with T1D to assess participants’ perceptions and preferences of each item. Based on results of the cognitive interviews, 11 candidate items (Supplementary Table 2) were selected for inclusion in the draft FoH screener, and participant instructions for completing the screener were drafted. The 11 items were rated on a 5-point Likert scale (1 for *strongly disagree* to 5 for *strongly agree*). As consistent with the literature, six items conceptually measured the avoidance behavior of FoH, and five items measured the worry component of FoH [8–13]. This report focuses on the validation phase to examine the reliability and validity of the new screener.

### Validation phase: study design and study population

We conducted a cross-sectional web-based (electronic) survey study to refine and potentially reduce the number of items in the draft FoH screener (11 items) and examine the reliability and validity of the new screener (Fig. 1).

Participants for the study were recruited from the online T1D Exchange Registry, a longitudinal registry that enrolls adults with T1D to collect information on diabetes management and outcomes [19, 20]. Key eligibility criteria included: age ≥ 18 years, diagnosed with T1D for at least 12 months, had glycated hemoglobin (HbA1c) measurements available within last six months, were residents of the United States, were fluent in written English, and were not pregnant at the time of the survey.

Potential study participants were invited via email with a link to the electronic consent form. After confirming eligibility and interest, participants completed the study-specific informed consent forms prior to completing the electronic survey. The study was reviewed and approved by the Western Institutional Review Board (WIRB® Protocol #20,202,118). Data were collected between August 10, 2020 – September 8, 2020.

### Measures

Participants reported demographic and diabetes-related health information, including gender, age, race/ethnicity, education, household income, past experience with hypoglycemia (defined as in need of assistance to recover in the last 12 months), and current use of diabetes devices (e.g., insulin pump, and continuous glucose monitoring [CGM]; yes/no). The following measures were also included in the survey, with more details described in Supplementary Table 3.

### Generalized anxiety disorder (GAD-7)

GAD-7 was a validated seven-item scale to assess generalized anxiety disorder and was reported on a 4-point Likert scale (0 = *not at all* to 3 = *nearly every day*) [21]. Responses to each item were summed to produce a total score ranging from 0 to 21, with higher scores indicating more severe symptoms of GAD.

### Patient health Questionnaire-8 (PHQ-8)

PHQ-8 was a validated eight-item scale that is used both as a diagnostic and severity measure for (likely) major depressive disorder. Similar to GAD, PHQ-8 was reported on a 4-point Likert scale (0 = *not at all* to 3 = *nearly every day*) [22]. Higher scores indicate more severe depression symptoms.

### Diabetes distress scale for adults with type 1 diabetes (T1-DDS)

T1-DDS was a validated measure that assesses diabetes-specific distress among adults with T1D. Patients were asked to rate the issues that have been a problem for them on a 6-point scale (1 – *not a problem* to 6 – *a very serious problem*). Three subscales were selected for this study: powerlessness (five items), management distress (four items), and hypoglycemia distress (four items) [23]. Higher scores indicate greater diabetes distress.

### Hypoglycemia fear Survey-II (HFS-II) short form

An 11-item short form of the HFS-II was used to measure the avoidance behavior and worry components of FoH. Items were rated on a 5-point Likert scale (0 = *never* to 4 = *almost always*) [9]. Higher scores indicate greater FoH.

### Statistical analysis

Descriptive statistics were conducted on the total survey sample to summarize participants’ demographic characteristics, diabetes management measures, diabetes-related complications and comorbidities, mental health measures, and experiences with hypoglycemia.

The total sample was then randomly split into two sub-samples (2:1 ratio) to examine the reliability and validity of the screener. Sample 1 was used to perform the initial analyses (described below), and Sample 2 was used to repeat the final factor analysis, and reliability and validity statistics were used to verify the findings from Sample 1. The two sub-samples were comparable (i.e., not significantly different) on key demographic and clinical characteristics.

For Sample 1, results of the initial analyses were examined at the individual item level to refine and reduce items if necessary. Item response distributions and inter-item correlations were examined. A series of exploratory factor analyses (EFA) followed to explore factor structure and factor loadings of each item. The final set of items were then examined for internal consistency (Cronbach’s alpha). Sum scores were calculated across the final items to generate the total screener score and domain scores (if necessary, based on factor analysis). Pearson correlations were used to examine concurrent validity between the screener score(s) and HFS-II short form scores, T1-DDS subscale scores, as well as GAD-7 and PHQ-8 scores. Multivariable regressions were then conducted to use the screener cut-off score categories (0 = low FoH, 1 = high FoH) to predict outcome measures relevant for diabetes management, including self-reported HbA1c, number of comorbid conditions, self-reported comfortable blood glucose range, as well as GAD-7, PHQ-8, and T1-DDS subscale scores.

Potential clinically meaningful cut-off scores were explored following an approach published by Fisher et al. [24] and Hajós et al. [15]. This approach was based on examining the score distributions in relation to other relevant diabetes outcomes and psychosocial measures (HbA1c, number of comorbidities, symptoms of anxiety, depression, diabetes distress, and hypoglycemia avoidance behavior). Through visual inspection of the distribution of scores, a cut-off score was identified by looking for clear and consistent separation points in the FoH screener score distributions that meaningfully differentiated diabetes and psychosocial outcomes (e.g., HbA1c > 7%, hypoglycemia unawareness [Gold score > = 4; Gold et al., 1994 [25]], moderate or severe anxiety symptoms).

The above multivariable regression analyses were conducted to validate the cut-off scores, and the proposed cut-off scores were then examined in multivariable regression models to predict key diabetes outcomes, such as HbA1c and number of comorbidities, adjusted for gender, age, duration of T1D, insulin pump use, and CGM use.

## Results

### Participant characteristics

A total of 620 participants responded to the invitation and completed the informed consent and electronic web-based survey, of which 28 were excluded for duplicate submissions. The final sample included 592 adults. Mean ± SD for age was 43.1 ± 15.3 years (Table 1). Mean duration of T1D was 24.1 ± 15 years. The majority of the participants were female (66.7%) and mean self-reported HbA1c was 7.1% ± 1.2%. Approximately 30% of participants reported at least one severe hypoglycemic episode (defined as in need of assistance to recover) in the last 12 months. Impaired hypoglycemia awareness (Gold score > = 4; Gold et al., 1994 [25]) was reported by 33.4% of the patients (Table 1).

**Table 1:** Patient characteristics

Variables	Number of patients	Mean (SD) or percentage (%)
Age, years	592	43.1 (15.3)
Duration of T1D, years	592	24.1 (15)
Self-reported HbA1c, %	592	7.1 (1.2)
Female, %	395	66.7%
Race, %		
American Indian/Alaskan Native	6	1.0%
Asian	13	2.2%
Black or African American	24	4.1%
Native Hawaiian or other Pacific Islander	2	0.3%
Other	20	3.4%
White	542	91.6%
Hispanic or Latino	31	5.2%
Insulin pump users, %	305	51.5%
Blood glucose monitoring, %		
BGM only	155	26.2%
CGM user	424	71.6%
Potentially impaired hypo awareness (score > = 4)		
Aware of hypo (1–3)	394	66.6%
Impaired awareness (4–7)	198	33.4%
Severe low blood sugar in the past 12 months	178	30.1%
Health insurance, %		
Private insurance	446	75.3%
Public insurance	129	21.8%
No insurance	10	1.7%
Don’t know/do not wish to answer	7	1.2%
Blood sugar levels to begin treating a low	591	71.1 (10)
Most recent severe low blood sugar occurrence		
Less than 1 year ago	182	30.7%
1–2 years ago	60	10.1%
2–5 years ago	57	9.6%
More than 5 years ago	117	19.8%

BGM, blood glucose monitoring; CGM, continuous glucose monitoring; HbA1c, glycated hemoglobin; SD, standard deviation; T1D, type 1 diabetes

### Screener reliability and validity

#### Exploratory factor analyses and correlation

Sample 1 (n = 397) was used for initial exploration and item reduction. In EFA using ProMax rotation, Sample 1 data showed low (0.3) factor loadings on two items (Supplementary Table 2). Considering both clinical relevance and screener content, these two items were removed from the final screener draft, resulting in nine items (Table 2). The EFA analysis showed a two-factor structure, with six items loading on a worry domain (factor loadings 0.624–0.909), and three items loading on an avoidance behavior domain (factor loadings 0.563–0.905).

**Table 2:** Two-factor exploratory factor loadings with Promax rotation

FoH screener items	Factor 1	Factor 2
I am afraid of having a low blood sugar when I am sleeping	0.712	
I am afraid of having a low blood sugar when no one is around to help me	0.909	
I am afraid of passing out due to a low blood sugar	0.889	
I am afraid of having a low blood sugar when I am out in public	0.640	
I am afraid of having a low blood sugar when I am driving	0.624	
I am afraid that I won’t catch and respond to a low blood sugar before it is too late	0.772	
I eat a lot more than I really need to avoid having a low blood sugar		0.696
I limit my physical activity to avoid having a low blood sugar		0.563
I keep my blood sugars high to avoid having a low blood sugar		0.905

FoH, fear of hypoglycemia. Items with factor loadings below 0.3 were not shown (Item: I make sure I have someone with me when I go out to avoid having a low blood sugar; Item: I eat a lot more often than I really need to avoid having a low blood sugar). All items were rated on a 5-point scale (1 – Strongly Disagree to 5 – Strongly Agree); Item scores were summed to produce worry and behavior subscale scores, and a total score. Higher scores indicate greater FoH.

The nine-item screener showed good internal consistency (total scale; Cronbach’s α = 0.88; Table 3) and was highly correlated (r = 0.71–0.75; Table 3) with the 11-item short form of the Hypoglycemia Fear Survey (“worry” and “behavior” subscales and total scores i.e., construct validity). Construct validity of the FoH screener was demonstrated with significant moderate positive correlations with depression (PHQ-8, r = 0.44), anxiety (GAD, r = 0.47), and Diabetes Distress Subscales (powerlessness, management distress, and hypoglycemia distress) (r = 0.49–0.66; Table 3). Additionally, multivariable regression analysis was conducted separately using continuous screener scores and categories based on the cut-off scores. Both the analyses showed that higher FoH screener scores, particularly for the avoidance behavior domain, were associated with higher HbA1c values (regression coefficient, b = 0.15; P < 0.001) and higher number of comorbidities (b = 0.06; P < 0.05); participants with higher behavior domain scores were more comfortable with higher blood glucose levels (Table 4). Results of Sample 2 were similar to Sample 1, thereby confirming our findings.

**Table 3:** Correlation coefficients between screener scores and established PROs

	Screener scores		
	Total N = 397	Worry N = 397	Behavior N = 397
Reliability and scores distribution			
Cronbach’s alpha	0.88	0.90	0.75
Score, mean (SD)	26.3 (8.3)	19.1 (6.3)	7.3 (3.1)
**Construct validity**			
Screener – worry	0.95	–	–
Screener – behavior	0.75	0.49	–
HFS – worry	0.75	0.74	0.48
HFS – avoidance behavior	0.71	0.60	0.68
HFS – total	0.81	0.75	0.62
GAD-7 (general anxiety)	0.47	0.42	0.40
PHQ-8 (depression)	0.44	0.36	0.44
T1-DDS - hypo distress	0.66	0.66	0.42
T1-DDS - management distress	0.49	0.39	0.50
T1-DDS - powerlessness	0.54	0.49	0.44

GAD-7, generalized anxiety disorder; HFS, hypoglycemia fear survey; N, number of patients; PHQ-8, patient health questionnaire-8; SD, standard deviation; T1-DDS, diabetes distress scale for adults with Type 1 diabetes. Data presented as Cronbach’s alpha /mean for reliability and scores distribution and as mean / correlation coefficients for construct validity; all P-values < 0.001

**Table 4:** Multivariable regression analysis for continuous screener total and domains scores

	Screener-total		Screener-domains		
Outcome variables	b	R^2^	b - worry	b - behavior	R^2^
Self-reported HbA1c (%)	0.04***	0.15	0.00	0.15***	0.21
Number of comorbidities	0.03**	0.33	0.01	0.06*	0.33
Comfortable BG range – low	0.56***	0.06	0.17	1.57***	0.07
Comfortable BG range – high	1.02***	0.04	-0.32	4.58***	0.09
Depression (PHQ-8)	0.27***	0.22	0.14**	0.63***	0.25
Anxiety (GAD-7)	0.31***	0.26	0.25***	0.46***	0.27
T1-DDS - hypo distress	0.11***	0.43	0.13***	0.06**	0.44
T1-DDS - management distress	0.06***	0.30	0.02**	0.16***	0.35
T1-DDS - powerlessness	0.08***	0.31	0.07***	0.11***	0.31

b, unstandardized regression coefficient; BG, blood glucose; GAD-7, generalized anxiety disorder; HbA1c, glycated hemoglobin. PHQ-8, Patient Health Questionnaire-8; T1-DDS, Diabetes Distress Scale for Adults with Type 1 Diabetes. Adjusted for covariates: gender, age, duration of T1D, insulin pump use, and continuous glucose monitoring (CGM) use. The table shows the unstandardized regression coefficients (b) between the continuous screener scores and outcome variables, as well as adjusted R-square for each regression model. The unstandardized regression coefficients show the unit change in outcome variables providing one unit change in the predictor, i.e. total screener score and domain scores because the predictors were binary variables. Comfortable BG range (mg/dL) – low: 85 (40–250). Comfortable BG range (mg/dL) – high: 150 (90–600). *P < 0.05, **P < 0.01, ***P < 0.001

### Screener cut-off scores

By examining the score distributions in relation to other relevant diabetes outcomes and psychosocial measures, we observed natural and consistent separate points in the FoH screener score distributions where HbA1c levels, symptoms of anxiety and depression, hypoglycemia awareness, and past experiences with severe hypoglycemia events were meaningfully different. The following cut-off scores were suggested for total and domain scores: total score above 30 (i.e., high FoH 31–45, low FoH 9–30), worry domain score above 23, and behavior domain score above nine (Supplementary Table 4). About 35% of participants were classified as having high FoH based on the total score. A multivariable regression analyses was conducted using a cut-off score of 10–15 to indicate high FoH by the behavior domain. The model was also adjusted for gender, age, duration of T1D, insulin pump use, and CGM use. Results showed that the average HbA1c of participants who scored above the total score cut-off of 30 was 0.52% higher than those participants who scored below the total score cut-off (Supplementary Table 5). On an average, those with high FoH (by total score) tended to keep their blood glucose levels at 12.7 mg/dL higher than those with low FoH group. Similarly, participants with high FoH (by the behavior domain), kept their blood glucose levels at 24.9 mg/dL higher than those with low FoH. The total and domain cut-off scores were also significantly associated with the number of comorbidities, i.e., depression, anxiety, and diabetes distress (Supplementary Table 4).

## Discussion

We developed and validated a screener for FoH in adults with T1D. The nine-item screener demonstrated good validity and internal consistency, strong correlations with a well-established FoH measure, and moderate positive correlations with related (yet distinct) psychosocial instruments, as well as associations with meaningful diabetes outcomes. The new screener supports a two-factor structure that is consistent with the literature on FoH covering both worries and avoidance behaviors related to hypoglycemia. It is important to note that the avoidance behavior domain demonstrated significant associations with higher HbA1c values, underscoring clinical relevance. Based on the domain scores and cut-offs, clinicians might gain insight into the level of severity and specific areas for targeted intervention to address patients’ concerns about FoH. The FoH screener can help to stimulate clinical conversations around the emotional impact of hypoglycemia and quantify the level of fear that may indicate a need for a referral for further assessment and treatment. Given that impaired awareness of hypoglycemia can also be a concern for developing severe hypoglycemic events [3], it would be a helpful if HCPs can routinely assess impaired awareness of hypoglycemia along with FoH, especially for individuals who have a low FoH score along with a Gold score ≥ 4.

### Strength and limitations

Our goal was to develop a valid, short, and actionable FoH screener for clinical use. One limitation is that the FoH screener was validated only in an adult population with T1D. Future research should examine the possibility to extend the screener for use among adults with T2D. The participants included in this survey were recruited from the T1D Exchange Registry that includes adults with relatively low HbA1c levels (though the screener may, in fact, be quite useful to identify FoH in adults with T1D who have higher HbA1c levels.)

## Conclusions

This screener can fill the gap in diabetes psychosocial care by providing a valid, short, and actionable tool for HCPs to utilize in clinical practice. The screener is a tool to initiate conversations and identify problematic FoH so that providers can assess the sources of their patients’ fears and make necessary treatment decisions or referrals.

## Electronic supplementary material

Below is the link to the electronic supplementary material.

Supplementary Material 1

## Data Availability

The datasets used and/or analyzed during the current study are available from the corresponding author on reasonable request.

## List of Abbreviations

FoH: Fear of Hypoglycemia
HFS-II: Hypoglycemia Fear Survey-II
HCS: Hypoglycemic Confidence Scale
HABS: Hypoglycemic Attitudes and Behavior Scale
FH-15: Fear of Hypoglycemia Scale
QSFH: Quick Screening for Fear of Hypoglycemia Instrument
HCPs: Healthcare providers
T1D: Type 1 diabetes
ADA: The American Diabetes Association
HbA1c: Glycated hemoglobin
CGM: Continuous glucose monitoring
GAD-7: Generalized Anxiety Disorder
PHQ-8: Patient Health Questionnaire-8
T1-DDS: Diabetes Distress Scale for Adults with Type 1 Diabetes
EFA: Exploratory factor analyses
BGM: Blood glucose monitoring
SD: Standard deviation
