# Supplementary material for: Development and validation of fear of hypoglycemia screener: results from the T1D exchange registry
Source: J Patient Rep Outcomes. 2023 May 9;7:43. doi: 10.1186/s41687-023-00585-9 (PMC10169988; doi:10.1186/s41687-023-00585-9)
Supplement: Supplementary file 1 — Supplementary Material 1 [file 41687_2023_585_MOESM1_ESM.docx]

**Supplementary Tables**

**Supplementary Table 1** Summary of items and recommendation for FoH screener: development phase

| Theme/item | Summary | Participant endorsement* | | Recommendation |
| --- | --- | --- | --- | --- |
|  |  | Least | Most |  |
| ***Theme 1: Fear being alone***   1. I am worried about no one being around to help me during a low blood sugar 2. I am confident that I can avoid serious problems due to low blood sugar when I'm alone | - Relevant theme to FoH; participants had varied responses to item 1 and 2 - Some had minor issues with the language “confident” and “serious problem” | 4 | 4 | Keep |
| ***Theme 2: Avoiding being alone***   1. To avoid low blood sugar and how it affected me, I made sure I had someone with me when I go out 2. To avoid low blood sugar and how it affects me, I have people check on me several times during the day or night | - Some felt this theme was repetitive to theme 1 - Some felt that “day” vs “night” were different experiences; some felt that “have people check on me” didn’t apply | 5 | 2 | Keep 3  Remove 4 |
| ***Theme 3: In public***   1. I am concerned that I might pass out in public due to a low blood sugar episode 2. I am terrified that I might pass out in public due to a low blood sugar episode | - Many participants felt that “concerned” was a more appropriate wording; however, a few participants felt that “terrified” better captured their degrees of fear | 3 | 3 | Remove the word “episode” throughout to be consistent  Remove 5  Keep 6 |
| ***Theme 4: Social situation***   1. I avoid social situations due to fear of having a low blood sugar episode | - Relevant theme to FoH - Many participants would rather over-prepare than avoid social situations | 6 | 6 | Remove |
| ***Theme 5: Sleep***   1. I feel afraid that I could have a serious low blood sugar episode when I'm asleep 2. I am confident that I can stay safe from serious problems with low blood sugar when sleeping | - Overwhelming endorsement from participants - Participants can feel both confident but still fearful of hypoglycemia during sleep | 0 | 14 | Keep |
| ***Theme 6: Travel***   1. I am afraid of traveling for fear of low blood sugar | - The language “traveling” covered too broad and diverse experiences across participants - Participants’ experiences with hypoglycemia during traveling varied greatly | 6 | 4 | Remove |
| ***Theme 7: Physical activity***   1. To avoid low blood sugar and how it affects me, I limit my physical activity 2. I am confident that I can stay safe from serious problems with low blood sugar during physical activity | - Participants found this theme very relevant to FoH - Some had issues with the wording “confident” and “serious problems” | 1 | 9 | Keep 11  Remove 12 |
| ***Theme 8: Keep BG high***   1. To avoid low blood sugar and how it affects me, I keep my blood sugar higher than usual in social situations 2. To avoid serious problems with low blood sugar, I tend to keep my blood sugars higher than I probably should 3. To avoid serious problems with low blood sugar, I tend to keep my blood sugars higher than what my HCP recommends to avoid long-term complications | - Some found the wordings in item 14 and item 15 unclear - Most participants found the theme relevant but responses to specific items differed across individuals; fewer people identified strongly with item 15 | 0 | 7 | Keep 13, 14  Remove 15 |
| ***Theme 9: Overtreat***   1. To avoid serious problems due to low blood sugar, I eat a lot more than I really need to 2. To avoid serious problems due to low blood sugar, I eat a lot more often than I really need to | - Relevant theme to FoH, however, language may not encompass all over-treating behaviors - More participants preferred 16 than 17 | 2 | 4 | Keep both, space the items out in the survey |
| ***Theme 10: Injury***   1. Because my blood sugar could go low, I am worried about accidently injuring myself or others | - Inconsistent understanding on what “injuring myself or others” encompasses - Some participants questioned the necessity of this item compared to other themes | 7 | 0 | Remove |
| ***Theme 11: Hypoglycemia unawareness***   1. I feel that I don’t notice the warning signs of low blood sugar as well as I used to 2. I am confident that I can catch and respond to low blood sugar before my blood sugar gets too low | - All participants found the theme relevant; more participants identified with 19 than 20 - Minor language issues on “as well as I used to” in 19 and “too low” in 20 | 3 | 13 | Keep |
| ***Other themes***   1. Because my blood sugar could go low, I am worried about difficulty thinking clearly when responsible for others 2. I feel that I can't ever be safe from the possibility of a serious low blood sugar episode 3. I am worried about passing out due to low blood sugar | - Many participants felt that 21 was more relevant to FoH that 22 and 23, but 21 still somewhat overlapped with 18 - Some had issues with “serious low” in 22 | 2 | 1 | Keep 21  Remove 22  Keep 23 (which covers the content in item 5) |

BG, blood glucose; FoH, fear of hypoglycemia; HCP, healthcare providers. *Participant endorsement was assessed by two questions at the end of the interview: “what were the top three useful items or themes?” and “what were the least three useful items or themes?”

**Supplementary Table 2** List of FoH screener items (11 items): developmental phase

| I am afraid of having a low blood sugar when I am sleeping |
| --- |
| I am afraid of having a low blood sugar when no one is around to help me |
| I am afraid of passing out due to a low blood sugar |
| I am afraid of having a low blood sugar when I am out in public |
| I am afraid of having a low blood sugar when I am driving |
| I am afraid that I won’t catch and respond to a low blood sugar before it is too late |
| I eat a lot more than I really need to avoid having a low blood sugar |
| I limit my physical activity to avoid having a low blood sugar |
| I keep my blood sugars high to avoid having a low blood sugar |
| I make sure I have someone with me when I go out to avoid having a low blood sugar |
| I eat a lot more often than I really need to avoid having a low blood sugar |

FoH, fear of hypoglycemia

**Supplementary Table 3** Details of the outcome measures

| **Outcome measures** | **Definition** |
| --- | --- |
| Participant characteristics | Participants self-reported on demographic and diabetes-related health information, including gender, age, race/ethnicity, education, household income, insurance type, living situation (alone or with others), duration of disease, past experience with hypoglycemia, current diabetes devices used, type of diabetes care providers, frequency of clinic visits, diabetes-related complications, and comorbidities. |
| Generalized Anxiety Disorder (GAD-7) | The GAD-7 anxiety scale was developed to diagnose and assess severity of generalized anxiety disorder. Participants are asked to report the frequency at which they experienced seven symptoms of anxiety over the past two weeks on a 4-point Likert scale (0 = *not at all* to 3 = *nearly every day*). Responses to each item were summed to produce a total score (range: 0–21) with higher scores indicating more severe anxiety. Anxiety severity is classified into four categories based on the GAD-7 total score: minimal (0–4), mild (5–9), moderate (10–14), and severe (15–21) (Spitzer et al., 2006). |
| Patient Health Questionnaire-8 (PHQ-8) | The PHQ-8 is a patient-reported outcome measure that is used both as a diagnostic and severity measure for depressive disorders in clinical studies. Patients are asked to report the frequency at which they experienced eight symptoms of depression over the past two weeks on a 4-point Likert scale (0 = *not at all* to 3 = *nearly every day*). Responses to each item are summed to produce a total score (range: 0 – 24) with higher scores indicating more severe depression. Depression severity is classified into five categories based on the PHQ-8 total score: none (0–4), mild (5-9), moderate (10–14), moderately severe (15–19), and severe (20–24). A score ≥10 is indicative of major depression (Kroenke & Spitzer, 2002). |
| Diabetes Distress Scale for Adults with Type 1 Diabetes (T1-DDS) subscales: Powerlessness, Management Distress, Hypoglycemia Distress. | The T1-DDS is a 28-item patient-reported outcome measure that assess diabetes distress among adults with T1D. Patients are asked to rate the issues that have been a problem for the on a six-point scale (1 = *not a problem* to 6 = *a very serious problem*). Three subscales are selected for this study: powerlessness (5 items), a broad sense of feeling discouraged about diabetes; management distress (4 items), disappointment with one’s own self-care efforts; and hypoglycemia distress (4 items), concerns about severe hypoglycemic events. Subscale scores are calculated as the mean item score, higher score indicates higher diabetes distress (Fisher et al., 2015). |
| Hypoglycemia Fear Survey-II (HFS-II) short form | The HFS-II short form is an 11-item patient-reported outcome measure derived from the 33-item HFS-II. The HFS-II short form assesses patients’ behavior (six items) and worry (five items) related to hypoglycemia in the past six months. Items are rated on a 5-point Likert scale (0 = *never* to 4 = *almost always*). Mean item scores are calculated to produce HFS-behavior (HFS-B) and HFS-worry (HFS-W) subscale scores, and a total score. Higher scores indicate greater fear of hypoglycemia (Grabman et al., 2017). |
| Fear of Hypoglycemia Screener | All candidate screener items resulted from the HCP and patient interviews. They included 11 items rated on a five-point scale (1 = *strongly disagree* to 5 = *strongly agree*). Final scoring algorithm will be examined during data analysis. |

GAD-7, generalized anxiety disorder; HCP, healthcare providers, HFS, hypoglycemia fear survey; PHQ-8, Patient Health Questionnaire-8; T1-DDS, Diabetes Distress Scale for Adults with Type 1 Diabetes.

**Supplementary Table 4** Screener cut-off scores

|  | **n** | **%** |
| --- | --- | --- |
| **Total Score: 30** |  |  |
| **Screener - total** |  |  |
| High FoH (31–45) | 137 | 34.5% |
| Low FoH (9–30) | 260 | 65.5% |
| **Worry subscale: 23** |  |  |
| **Screener - worry** |  |  |
| High FoH worry (24–30) | 105 | 26.4% |
| Low FoH worry (6–23) | 292 | 73.6% |
| **Behavior subscale: 9** |  |  |
| **Screener - behavior** |  |  |
| High FoH behavior (10–15) | 92 | 23.2% |
| Low FoH behavior (3–9) | 305 | 76.8% |

FoH, fear of hypoglycemia.

**Supplementary Table 5** Regression coefficients between continuous outcome variables and screener total / screener domain cut-off scores respectively

|  | **Screener-total cut-off score group** | | **Screener-domains cut-off score group** | | |
| --- | --- | --- | --- | --- | --- |
| **Outcome variables** | **b** | **R^2^** | **b - worry** | **b - behavior** | **R^2^** |
| Self-reported HbA1c (%) | 0.52*** | 0.11 | 0.05 | 0.72*** | 0.13 |
| Number of comorbidities | 0.36* | 0.32 | 0.09 | 0.23 | 0.31 |
| Comfortable BG range - low | 6.24** | 0.03 | 0.92 | 7.32** | 0.03 |
| Comfortable BG range - high | 12.67* | 0.03 | 1.35 | 24.91*** | 0.06 |
| Depression (PHQ-8) | 3.38*** | 0.15 | 1.89** | 3.97*** | 0.20 |
| Anxiety (GAD-7) | 0.31*** | 0.26 | 0.25*** | 0.46*** | 0.27 |
| T1-DDS - hypo distress | 1.50*** | 0.30 | 1.41*** | 0.69*** | 0.32 |
| T1-DDS - management distress | 0.78*** | 0.22 | 0.36** | 0.98*** | 0.28 |
| T1-DDS - powerlessness | 1.07*** | 0.21 | 0.77*** | 0.86*** | 0.23 |

b, beta coefficient; BG, blood glucose; PHQ-8, Patient Health Questionnaire-8; T1-DDS, Diabetes Distress. Cut-off scores were entered in the regression model as binary variables (e.g., 0 = low FoH, 1 = high FoH). unstandardized Scale for Adults with Type 1 Diabetes. *P<0.05, **P<0.01, ***P<0.001. Regression model was controlled for gender, age, duration of T1D, insulin pump use, and CGM use.
